# Hacking Extracellular Vesicles: Using Vesicle-Related Tags to Engineer Mesenchymal Stromal Cell-Derived Extracellular Vesicles

**DOI:** 10.3390/pharmaceutics17111435

**Published:** 2025-11-06

**Authors:** Gabriele Scattini, Giulia Pianigiani, Stefano Capomaccio, Maria Rachele Ceccarini, Samanta Mecocci, Laura Musa, Luca Avellini, Olimpia Barbato, Antonello Bufalari, Patrizia Casagrande Proietti, Rodolfo Gialletti, Alessia Sulla, Tommaso Beccari, Luisa Pascucci

**Affiliations:** 1 Department of Veterinary Medicine, University of Perugia, 06126 Perugia, Italy; gabriele.scattini@unipg.it (G.S.); stefano.capomaccio@unipg.it (S.C.); sethy46@yahoo.it (S.M.); luca.avellini@unipg.it (L.A.); olimpia.barbato@unipg.it (O.B.); antonello.bufalari@unpg.it (A.B.); patrizia.casagrandeproietti@unipg.it (P.C.P.); rodolfo.gialletti@unipr.it (R.G.); alessia.sulla@dottorandi.unipg.it (A.S.); 2 Department of Medicine and Surgery, University of Perugia, 06129 Perugia, Italy; 3 Department of Pharmaceutical Sciences, University of Perugia, 06123 Perugia, Italy; mariarachele.ceccarini@unipg.it (M.R.C.); tommaso.beccari@unipg.it (T.B.)

**Keywords:** Mesenchymal Stromal Cells, extracellular vesicles, transfection, CD63, Syntenin, TSG101, Palmitoylation, EVs engineering, GFP

## Abstract

**Background/Objectives**: Extracellular Vesicles (EVs) have shown great promise as diagnostic and therapeutic tools, as well as pharmacological nanocarriers. Various strategies are being explored to develop EVs for monitoring, imaging, loading with pharmacological agents, and surface decoration with tissue-specific ligands. EVs derived from Mesenchymal Stromal Cells (MSC-EVs) are of particular interest both as therapeutics *per se* and as natural nanocarriers for the targeted delivery of biotherapeutics. **Methods**: In this study, we investigated the ability of different tags to deliver a reporter protein into canine MSC-EVs with the aim of identifying the most effective endogenous loading mechanism. To this aim, canine MSCs were engineered to express the Green Fluorescent Protein (GFP) fused to CD63, Syntenin-1, TSG101, and the palmitoylation signal of Lck, which were expected to promote GFP incorporation into EVs. Overexpression of tagged GFP in canine MSCs was confirmed by Western blotting and examined by confocal microscopy and transmission electron microscopy to map intracellular localization. **Results**: All tags were able to deliver GFP into EVs. Syntenin-1 showed relatively high loading efficiency and secretion index but exhibited a diffuse localization pattern in the transfected cells. The palmitoylation signal showed low loading efficiency and localization specificity. TSG101 displayed a morphological pattern consistent with specific localization in endosomal structures, but its low expression level prevented further evaluations. Finally, CD63 showed the highest expression efficiency, as GFP-CD63 levels were approximately 5-fold higher than untagged GFP. **Conclusions**: In conclusion, CD63 emerged as the most suitable tag for canine MSC-EV engineering. Indeed, even if the secretion index favours Syntenin-1, CD63’s higher abundance in the lysate suggests its substantial post-secretion uptake. Further studies aimed at elucidating CD63’s specific contribution and identifying the domains involved in vesicle trafficking could provide valuable insights into EV bioengineering.

## 1. Introduction

In recent decades, the study of extracellular vesicles (EVs) as highly specific mediators of intercellular communication over short and long distances has developed rapidly, involving almost all biomedical fields and emphasizing their crucial role in various physiological and pathological conditions [1]. EVs are micro- and nanoparticles surrounded by a lipid bilayer and containing a variety of biological molecules, from nucleic acids to small metabolites. EVs are produced by virtually all cells, which use them to pack various cargoes and release them into the extracellular space. EVs possess intrinsic targeting characteristics, natural composition, resistance to harsh environmental conditions, reduced clearance, stability in the bloodstream, and the ability to cross biological barriers [2].

Currently, EVs are categorized into three main groups based on their mechanisms of biogenesis: exosomes (Ex), which arise from multivesicular bodies (MVBs), microvesicles (MVs), which bud directly from the plasma membrane, and apoptotic bodies (ABs), which originate from dying cells. EVs exert their functions by releasing cargo into target cells through membrane fusion or by interacting with surface receptors on target cells [3].

The growing interest in EVs stems from their potential in the biomedical field. Many studies have shown that they can be used as diagnostic markers for various diseases, from cancer to neurological disorders [4,5]. In addition, their potential use as nanocarriers for the release of therapeutic molecules is generating great excitement in the drug delivery field, which is rapidly moving from the micro- to the nanoscale [6]. By engineering suitable parental cells, it may be possible to transform them into natural bioreactors and produce EVs with known content and well-defined functions [7]. To this end, several research groups have explored strategies to engineer the surface of EVs with ligands that target the tissue and/or to modify their cargo. EVs used as carriers of small drugs showed high biocompatibility and a good pharmacokinetic profile in vivo [8]. More recently, EVs have also been investigated as carriers for large biomolecules such as proteins and RNA [9,10]. Protein-based therapies are gaining increasing relevance in healthcare because of their therapeutic potential. However, their clinical application is frequently limited by instability, short half-life, and immunogenicity, which can affect both safety and efficacy. In this context, nanoscale delivery systems such as EVs could represent a valuable strategy to overcome these limitations and enhance the therapeutic impact of protein-based approaches.

To date, only a few reports have described the successful loading of proteins into EVs. This suggests that the mechanisms regulating the transport and loading of biomolecules into EVs are still relatively unexplored and that new methods for tailoring EV cargo to the target tissue need to be developed. The endosomal sorting complex required for transport (ESCRT) system has been the main system investigated [11]. Alternative mechanisms were described by Trajkovic and colleagues, who reported ESCRT-independent, ceramide-mediated biogenesis of EVs [12]. Van Niel and colleagues described a different pathway in which CD63 regulates EV biogenesis without the involvement of ESCRT [13].

More recently, the number of research groups investigating biogenesis, transport, and internalization of EVs by cell engineering has greatly increased. Most of these studies use CD63 tagged with different reporter systems, such as Green Fluorescent Protein (GFP) [14], HaloTag [15], and pHluorin [16].

The choice of an appropriate donor cell is a key element in the development of genetically engineered EVs to be used as biocarriers. Ideally, the selected cell type should produce EVs that lack immunostimulatory activity, are stable in the bloodstream, and can preferentially reach the target. The use of Mesenchymal Stromal Cells (MSCs) appears both attractive and feasible. MSCs are a population of stromal cells whose therapeutic potential is being extensively studied in both human and veterinary medicine [17].

They can be isolated from various tissues and biological fluids. Adipose tissue is a strategic source of MSCs because it is abundant, easy to obtain, and rich in MSCs. Originally classified as stem cells, MSCs are now considered “medical signaling cells”, as their therapeutic effect is achieved mainly through the paracrine secretion of bioactive principles rather than through their engraftment and differentiation [18]. The paracrine activity of MSCs is largely mediated by the release of soluble factors and EVs [19,20,21,22]. Moreover, many studies have shown that MSCs can be safely used in animal models and clinical trials as well as in allogeneic recipients [23].

The present study aimed to engineer canine adipose-derived mesenchymal stem cells (c-Ad-MSCs) to promote the release of EVs carrying a defined protein cargo. The overarching objective is to explore the capacity of EVs to mediate molecular transport and to establish a framework for their potential functionalization with proteins of therapeutic interest. Specifically, we induced the overexpression of GFP tagged with molecules known to be concentrated in EVs based on specific motifs or localization signals, some associated with the lipid membrane and others with intra-luminal localization. The selected tags were CD63, Syntenin-1, TSG101, and the palmitoylation signal of Lck (Table 1).

Among these, CD63 appeared as the most effective tag for canine MSC-EV engineering, combining robust loading efficiency with a well-defined intracellular localization that supports controlled delivery.

The species-specific approach adopted in this study not only addresses the growing demand for innovative therapies in veterinary medicine but also provides a clinically relevant large-animal model that bridges the gap between preclinical rodent studies and human applications, thereby enhancing the translational potential of EV-based therapeutic strategies.

## 2. Materials and Methods

### 2.1. Isolation, Culture, and Characterization of Canine MSC

c-Ad-MSCs were isolated from the subcutaneous adipose tissue of a 5-year-old dog that had died from trauma and was referred to the OVUD (Veterinary University Hospital) of the University of Perugia. The adipose tissue was removed within 2 h after death with the consent of the owners, who made the cadaver available for teaching and research purposes. The tissue sample was washed three times with DPBS (Dulbecco’s Phosphate-Buffered Saline) supplemented with penicillin (100 U/mL), streptomycin (100 μg/mL), and amphotericin B (2.5 μg/mL) (Merck, Darmstadt, Germany). The tissue was then minced with sterile scissors and digested for 70 min in a 0.075% collagenase I solution (Worthington Biochemical Corp., Lakewood, NJ, USA) at 37 °C in a water bath with gentle agitation. After digestion, collagenase activity was blocked by the addition of DMEM supplemented with 10% fetal bovine serum (FBS). The stromal vascular fraction (SVF) obtained by collagenase digestion was centrifuged at 600× *g* for 10 min to remove the fat fraction and collagenase solution and seeded in cell culture flasks containing DMEM supplemented with 10% FBS, 100 U/mL penicillin, and 100 μg/mL streptomycin. After 48 h, the unattached material was removed. The medium was changed every 2–3 days, and the cells were passed when they had reached 80–90% confluence. Passage 3 c-Ad-MSCs were characterized using the International Society for Cell and Gene Therapy (ISCT) criteria to assess the ability to differentiate into adipocytes, osteoblasts, and chondroblasts and to confirm the expression of characteristic c-Ad-MSC surface markers. For trilineage differentiation, cells were seeded in 24-well plates, stimulated with differentiation media for 3 weeks, and then stained with Oil-O-Red, Alizarin, or Alcian Blue as described by Neupane and colleagues [24]. The immunophenotype of c-Ad MSCs was determined by FACS analysis (for CD14, CD29, CD44, CD45, CD90, and MHCII as described by Ivanovska and colleagues [25].

### 2.2. RNA Isolation and cDNA Synthesis

Total RNA was extracted from 3rd-passage c-Ad MSCs using Trizol and purified using the PureLink MiniKit (Invitrogen, Carlsbad, CA, USA) according to the manufacturer’s instructions. The isolated RNA was quantified with a NanoDrop spectrophotometer (Thermo Fisher Scientific, Waltham, MA, USA). The first strand of cDNA was then synthesized by reverse transcription of total RNA (1 μg per sample) using SuperScript Vilo IV (Thermo Fisher Scientific, Waltham, MA, USA) in a final volume of 20 μL (thermal cycling conditions: 25 °C for 10 min, 42 °C for 60 min, 85 °C for 5 min).

The sequences encoding CD63, TSG101, and Syntenin-1 were amplified by PCR with appropriate primers (Table 2) containing restriction sites for subsequent ligation. A high-fidelity TaqQ5 enzyme was used for amplification (New England Biolabs, Ipswich, MA, USA).

### 2.3. Plasmids

PCR products were separated by electrophoresis on a 1.2% agarose gel in TAE buffer. The amplicons of interest, corresponding to the expected size, were then excised from the gel and purified using the Zymoclean Gel DNA Recovery Kit (Zymo Research, Irvine, CA, USA). The purified PCR products were digested with HindIII/SalI (for CD63 and TSG101) and KpnI/SalI (for Syntenin-1) enzymes and ligated into pBlueScript II SK with T4 ligase (New England Biolabs, Ipswich, MA, USA). Chemically competent XL1-Blue strains were transformed with the ligation products, and Sanger sequencing was performed on both strands of the purified plasmids to confirm the identity of the amplified sequences, as described by Hedayati et al. [26].

The CD63, TSG101, and Syntenin-1 sequences were subcloned into pTag-GFP2-N (Evrogen, Moskva, Russian Federation) by removing the stop codon and linking to the GFP coding sequence in the SalI restriction site in frame.

### 2.4. MSC Transfection and Isolation of EVs

c-Ad-MSCs at passage 3 were seeded at 5 × 10^3^ cells/cm^2^ in DMEM supplemented with 10% FBS, penicillin (100 U/mL), and streptomycin (100 μg/mL). After 24 h, the cells were transfected with the desired plasmid using Lipofectamine Stem Transfection Reagent (Thermo Fisher Scientific, Waltham, MA, USA) at a 1:4 ratio, with 250 ng DNA/cm^2^, according to the manufacturer’s instructions. Transfection was performed in the absence of FBS, which was added 12 h later to restore the normal concentration (10%). Cells were incubated for 24–48 h before subsequent analysis.

To isolate EVs, the culture medium was removed, and the cells were washed with DPBS to remove serum proteins. The medium was replaced with DMEM supplemented with penicillin (100 U/mL) and streptomycin (100 μg/mL), and incubated for 48 h. The conditioned medium was harvested and centrifuged at 1000× *g* for 15 min at 4° C to remove detached cells and cell debris. The supernatant was subsequently ultracentrifuged at 100,000× *g* for 60 min at 4 °C; the EV pellet was resuspended in 100 μL DPBS. The protein content was quantified using the Bradford assay.

### 2.5. Confocal Microscopy of Living Cells

t-GFP cells were seeded in 96-well black/clear plates (Corning Inc., Corning, NY, USA) and transfected as previously described to study their localization in c-Ad-MSCs. After 48 h, the cells were stained with fluorescent probes to better localize the different intracellular compartments: ER-ID Red Assay (Enzo Life Science, Farmingdale, NY, USA) for the endoplasmic reticulum, Hoechst 33,342 for the nuclei, LysoView 405 (Biotium, Fremont, CA, USA) for lysosomes as well as for other acidic compartments in the cell, including early and late endosomes, MitoView 650 (Biotium, Fremont, CA, USA) for mitochondria. Images were acquired using a Zeiss LSM 800 confocal microscope (Carl Zeiss AG, Oberkochen, Germany) with a Plan Apochromat 63×/1.40 Oil DIC M27 objective. The excitation wavelengths were 488 nm for GFP, 353 nm for Hoechst and LysoView, 592 nm for ER-Red stainer, and 650 nm for Mitoview. The original images were 1024 × 1024 pixels in size, and the pixel size was 187 × 187 nm.

### 2.6. Immunoelectron Microscopy of Transfected c-Ad-MSCs

Transfected c-Ad MSCs were fixed in Karnovsky fixative modified solution (4% paraformaldehyde, 0.5% glutaraldehyde in 0.1 M phosphate buffer, pH 7.4). Cells were dehydrated up to 70% in a graded ethanol series and embedded in LR White resin (Agar scientific, Essex, UK). Ultrathin sections mounted on Formvar-coated nickel grids (Agar scientific, Essex, UK) were incubated in a blocking solution (2% BSA and 2% normal goat serum in DPBS) for 60 min. The anti-GFP antibody (Life Technologies Carlsbad, CA, USA) was incubated overnight at room temperature in a blocking solution. A 12 nm gold-conjugated anti-rabbit secondary antibody (Jackson ImmunoResearch, Baltimore, MD, USA) was used to detect the primary antibody. The grids were contrasted for 2 min in 2% uranyl acetate solution and observed using a Philips EM208 equipped with a digital camera.

### 2.7. Western Blotting

The expressions of t-GFP were detected by Western blotting in cell lysates and isolated EVs. Transfected cells and EVs were lysed in RIPA buffer, and 10 μg of total proteins were mixed with Laemmli buffer, heated at 95 °C for 5 min, and separated by SDS-PAGE (10% polyacrylamide, 120 V for 2 h). The proteins were transferred to a nitrocellulose membrane and blocked with 5% non-fat milk for 60 min. Membranes were incubated overnight at 4 °C with primary antibodies diluted in 5% non-fat milk with gentle shaking. Incubation with HRP-conjugated secondary antibodies was carried out for 60 min at room temperature. Immune complexes were detected using ImageQuant LAS 500 (Cytiva, Marlborough, MA, USA) after the addition of the ECL solution. The band area density was analyzed using ImageQuant TL software v. 8.2 (Cytiva, Uppsala, Sweden), and statistical analyses were performed in Jasp v. 0.95.1.0 (JASP Team, University of Amsterdam, Amsterdam, The Netherlands; https://jasp-stats.org, accessed on 1 August 2025) using an independent-samples *t*-test. Antibody list: anti-GFP diluted 1:7500 (A6455, Life Technologies, Carlsbad, CA, USA), anti-TSG101 diluted 1:500 (sc-7964, Santa Cruz Biotechnology, Santa Cruz, CA, USA), anti-Alix diluted 1:500 (sc-271975, Santa Cruz Biotechnology, Santa Cruz, CA, USA), anti-bTubulin diluted 1:10,000 (ab131205, Abcam, Cambridge, MA, USA), anti-mitofilin diluted 1:10 (ab109424, Abcam, Cambridge, MA, USA), goat anti-mouse IgG HRP conjugated diluted 1:10,000 (A90-116P, Fortislife, Boston, MA, USA), and goat anti-rabbit IgG HRP conjugated diluted 1:10,000 (A120-101P, Fortislife, Boston, MA, USA).

## 3. Results

### 3.1. Canine Cells Meet the ISCT Inclusion Criteria for MSCs

**Figure 1:**
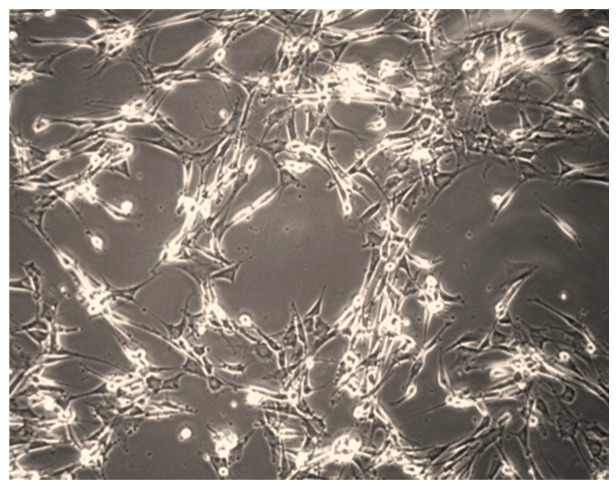
Fibroblast-like morphology of c-Ad-MSCs in culture. Contrast-phase microscopy (40× magnification).

c-Ad-MSCs were cultured and characterized according to the guidelines of the International Society for Cell and Gene Therapy [27]. They grew on plastic surfaces, exhibited a fibroblast-like morphology (Figure 1), were positive for the stromal markers CD29, CD44, and CD90 (as suggested for dogs in the relevant literature) and negative for CD14, CD45, and MHCII [28]. They also demonstrated the ability to differentiate into adipogenic, osteogenic, and chondrogenic lineages.

### 3.2. DNAs Encoding EVs Markers Show Complete Identity Compared to the Annotated Sequence

**Figure 2:**
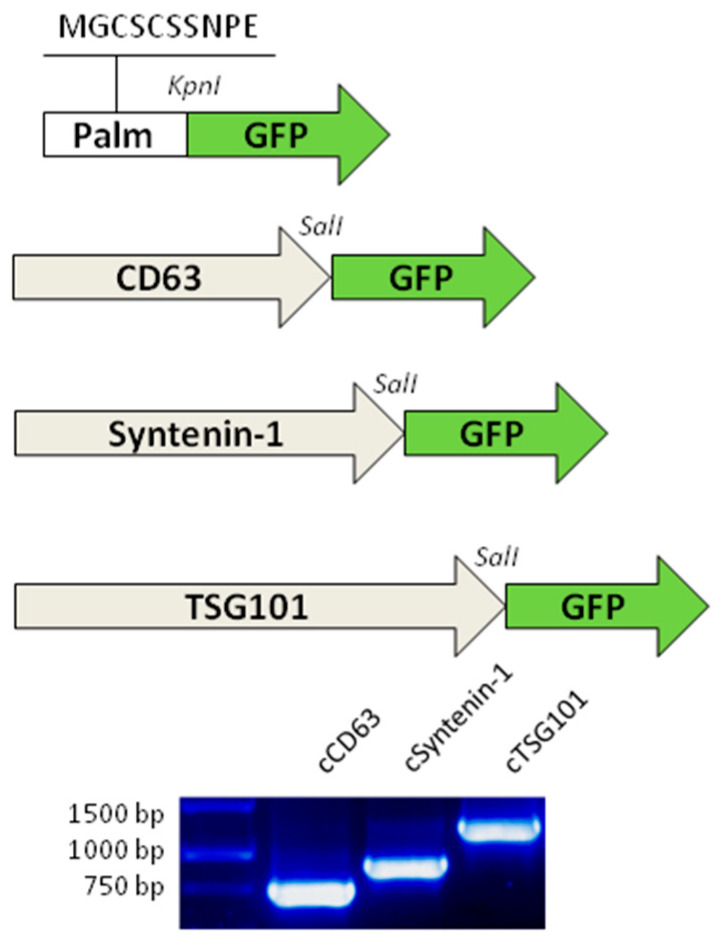
Schematic maps of the sequences encoding GFP-tagged proteins. **Top**: The sequence encoding the palmitoylation signal was ligated into the KpnI restriction site of pTagGFP2-N. cDNA from canine RNA was ligated into the SalI restriction site of pTagGFP2-N. **Bottom**: Gel electrophoresis of PCR products from the amplification of canine cDNA. Grey arrows: cDNA coding sequences. Green arrows: GFP coding sequence. White arrow: synthetic oligo for Lck palmitoylation motif. MGCSCSSNPE: amino acid sequence of Lck motif. KpnI and SalI restriction enzymes used for cloning process.

The cDNAs encoding CD63, Syntenin-1, and TSG101 were synthesized from the total RNA of c-Ad-MSCs by RT-PCR. The DNA fragments were cloned into plasmid vectors and sequenced to verify their identity (Figure 2). The sequencing results confirmed the 100% identity in the coding region compared to the NCBI predicted sequences (Cd63: XM_038678918.1; Syntenin-1: XM_852382.6; Tsg101: XM_038430665.1). In addition, the coding sequence of a splice variant of Syntenin-1, lacking glutamine at position 81, was also cloned (Syntenin ΔQ81: XM_038441228.1).

### 3.3. Transfected Canine MSCs Express GFP

**Figure 3:**
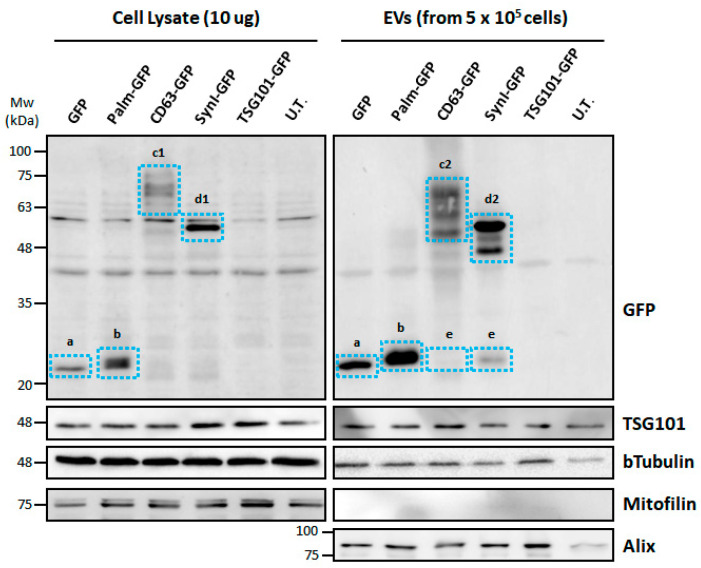
Western blotting for the detection of GFP-tagged proteins in c-Ad-MSC lysate and c-Ad-MSC-derived EVs. The anti-GFP immunoblotting assay in the upper panels shows the different molecular sizes of the chimeric proteins. a: untagged GFP (27 kDa); b: palmitoylated GFP (29–30 kDa); c1: CD63-GFP (65–75 kDa), polyubiquitylated forms; c2: CD63-GFP (60–75 kDa), polyubiquitylated forms; d1: Syn-GFP (60 kDa), full length; d2: Syn-GFP (50–60 kDa), full length and clavated or short form; e: untagged/cleaved GFP. Control positive markers of EVs: TSG101 (49 kDa) and Alix (95 kDa). Control negative markers of EVs: Mitofilin (75 kDa). Loading control: b-Tubulin (50 kDa). Palm-GFP: Palmitoylated GFP, Syn-GFP: Syntenin-1-GFP, UT: not transfected, Mw: molecular weight.

Untagged GFP and palmitoylated GFP showed the expected molecular weight (27 kDa and 29–30 kDa, respectively). CD63-GFP showed several bands with different molecular weights. The theoretical size of the chimeric protein is 54.8 kDa (516 amino acids), but due to the abundant ubiquitynation of CD63, its apparent molecular weight increases. A small amount of untagged GFP was also detected in CD63-GFP-transfected cells. Syn-GFP (Syntenin-1-GFP) showed a strong signal at the expected weight of 60 kDa (550 amino acids); as with CD63-GFP, a small amount of untagged GFP was also present. TSG101-GFP expression was low, as confirmed by Western blotting of cell lysates from c-Ad-MSCs (Figure 3).

### 3.4. EVs from Transfected MSCs Contain GFP-Tagged Proteins

Western blotting on EVs showed the presence of both tagged (tGFP) and untagged GFP. As in cell lysates, GFP and palmitoylated GFP showed a single band at the expected weight. For CD63-GFP, three discrete high-molecular-weight bands were detected, compatible with different ubiquitinated forms, while a weak signal was observed at the expected weight (55 kDa) and another at 27 kDa corresponding to untagged GFP. Syn-GFP showed a strong signal at the expected molecular weight. In addition, several bands were detected that were brighter than the full-length bands. They could indicate the expression of clivated proteins (Figure 3).

### 3.5. Palmitoylation Signal, CD63, and Syntenin-1 Confer Different Loading Efficiencies in EVs

**Figure 4:**
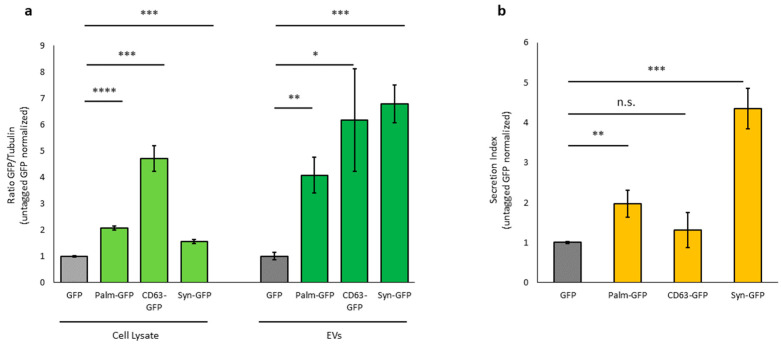
Ratio between secreted and intracellular tagged GFP. (**a**)—Relative amount of GFP content in cell lysates of c-Ad-MSC overexpressing tagged proteins and GFP content in isolated EVs, calculated based on Western blotting. Tagged GFP was normalized to untagged GFP. GFP in cells and EVs was normalized to bTubulin. (**b**): Graphical representation of the ratio between secreted GFP and intracellular GFP. (Palm-GFP: palmitoylated GFP, Syn-GFP: Syntenin-1-GFP). *p*-value * *p* < 0.05; ** *p* < 0.01; *** *p* < 0.001; **** *p* < 0.0001; n.s. > 0.05.

The loading efficiency of the reporter protein in EVs was estimated from the ratio of GFP-EVs and GFP cell lysates. The total amount of differentially labelled tGFP, both intracellular and secreted, was higher than that of untagged GFP. Palm-GFP was approximately 2-fold higher, CD63-GFP 5-fold higher, and Syn-GFP 1.6-fold higher than untagged GFP in cell lysates and 4-fold higher for Palm-GFP, 6-fold for CD63-GFP, and 7-fold higher for Syn-GFP in EVs (Figure 4a). The ratio between intracellular tGFP and tGFP released from EVs showed different efficiencies depending on the tag. Palm-GFP was released at levels 2-fold higher, Syn-GFP 4.3-fold higher, and CD63-GFP 1.3-fold higher than untagged GFP (Figure 4b). These data confirm that a protein of interest can be incorporated into EVs more efficiently when tagged with EV-associated proteins.

### 3.6. tGFP Shows Different Intracellular Localizations

**Figure 5:**
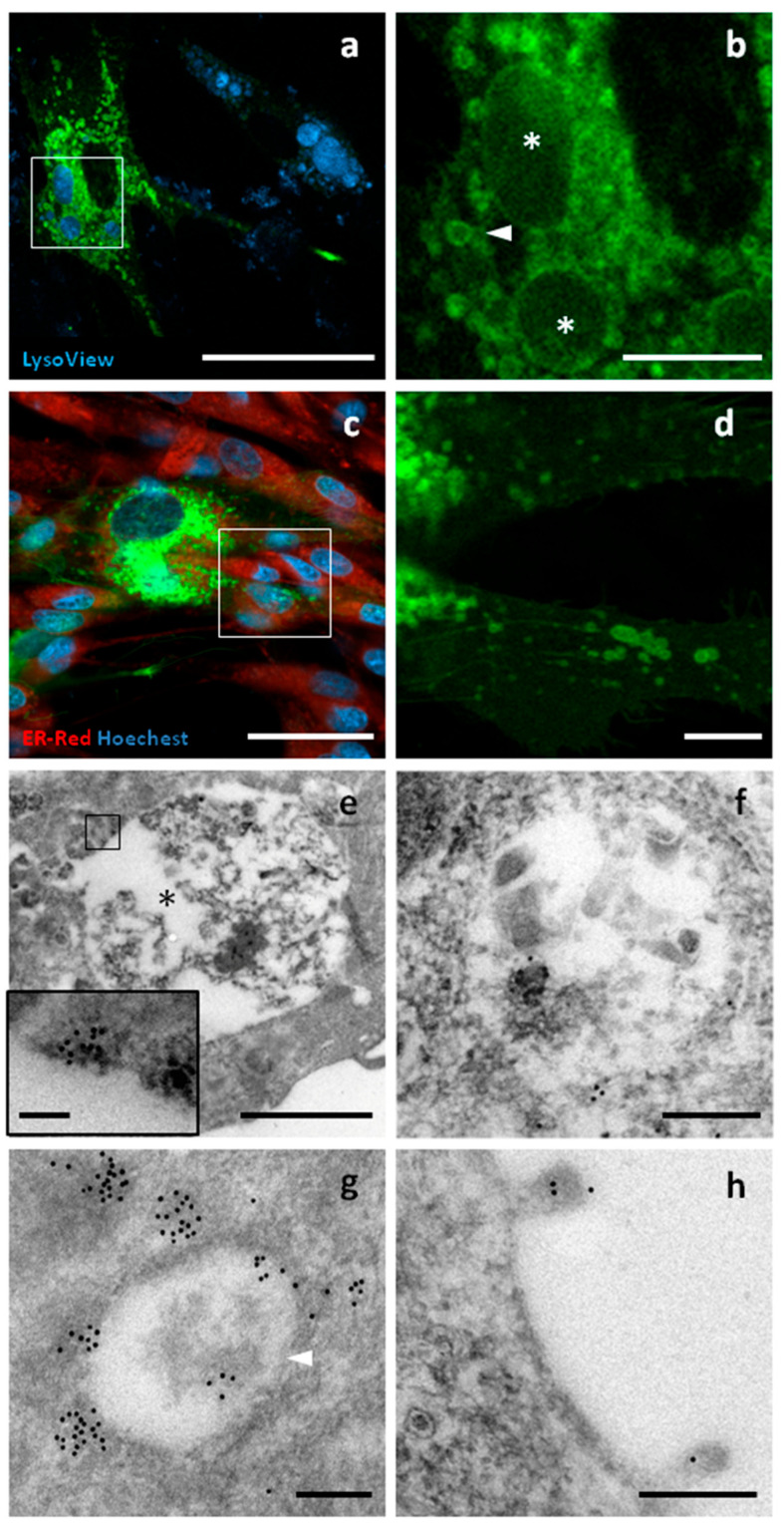
CD63-GFP localization. Confocal and TEM micrographs of transfected c-Ad-MSCs overexpressing CD63-GFP. Cells were labelled for lysosomes and other acidic compartments, including early and late endosomes (Lysoview—dark blue), for nuclei (Hoechst—light blue), and for endoplasmic reticulum (ER-ID Red Assay—red). (**a**) CD63–GFP displayed a focal distribution (green) involving compartments of different sizes. (**b**) A higher magnification view of the boxed region in (**a**) reveals large (asterisks) and small (arrowheads) compartments showing CD63–GFP localization. (**c**) A weak surface distribution of CD63–GFP was also observed (green). (**d**) A higher magnification view of the boxed region in (**c**). In (**e**), a large compartment is shown (asterisk), mainly reactive along the boundary. The inset shows a magnified view of the area outlined by the square. In (**f**), a late endosome with positive vesicles is visible in the lumen, as shown by the presence of black gold dots. In (**g**), a small endosome with gold particles mainly observed at the periphery (white arrowhead). In (**h**), positive vesicles are emerging from the cell surface. Scale bars: (**a**–**c**) 50 μm, (**b**–**d**) 10 μm, (**e**) 2 μm, (**e**) (inner panel) 100 nm, (**f**–**h**) 200 nm.

**CD63** belongs to the tetraspanin family, a group of transmembrane proteins with four α-helices and two extracellular domains. GFP was fused to the C-terminus of canine CD63, a region with cytoplasmic topology. CD63 is usually associated with the cell surface, as well as with lysosomes, endosomes, multivesicular bodies, and EV membranes. Using confocal microscopy, strong fluorescence was observed in intracellular compartments ranging from 300 nm to 5 μm in size. The largest compartments were mainly positive at the periphery and were strongly stained by LysoView. A weak signal was detected at the level of the cell membrane, where surface structures such as nanotubes and budding vesicles were observed. GFP signal was not detected in the nucleus or the endoplasmic reticulum. Similar substructures were also observed by TEM, with the largest having a diameter of up to 5 μm and being heterogeneous in terms of electron density and structure. Immunopositivity for GFP was found in the peripheral regions of these organelles. The smaller structures were between 300 and 800 nm in size and showed a more homogeneous structure and electron density. GFP localization appeared to be focally distributed around and within these compartments (Figure 5).

**Figure 6:**
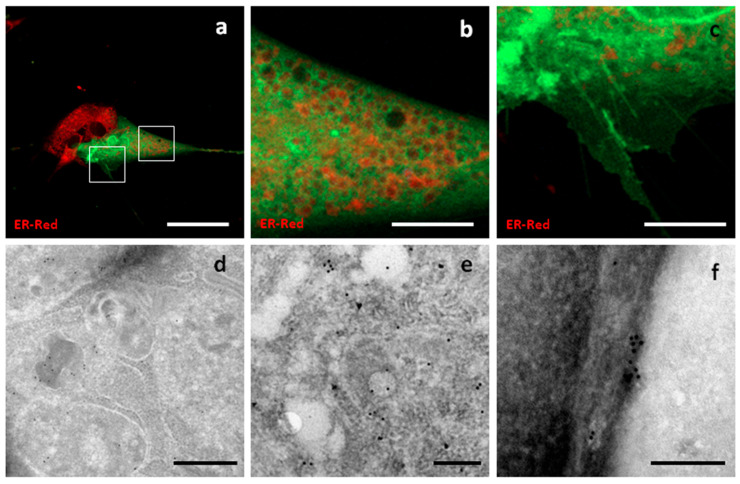
Localization of Syntenin-1-GFP. Confocal and TEM micrographs of transfected c-Ad MSCs overexpressing Syn-GFP. Syn-GFP showed a diffuse distribution in the cytoplasm (**a**), at the periphery of intracellular vesicular compartments (**b**), and on cell surface protrusions (**c**). Panels (**b**) and (**c**) show higher magnification views of the boxed regions in (**a**). Immunogold labelling showed a diffuse distribution in the cytoplasm (**d**,**e**) and a superficial focal distribution (**f**). Scale bars: (**a**) 50 μm, (**b**,**c**) 10 μm, (**d**) 500 nm, (**e**,**f**) 200 nm.

**Syntenin-1,** also known as **SDBCP**, is a cytoplasmic multifunctional adaptor involved in many biological processes. The sequence encoding canine Syntenin-1 was cloned into a GFP-encoding vector to obtain a construct expressing GFP fused to the C-terminus of Syntenin-1. A highly variable distribution of fluorescence was observed, with diffuse cytoplasmic localization being the most common pattern. In addition, Syn-GFP was observed in the nucleus, at the nuclear membrane, around intracellular compartments resembling endosomal elements, on the plasma membrane, and on surface structures resembling nanotubes and nascent microvesicles (Figure 6).

**Figure 7:**
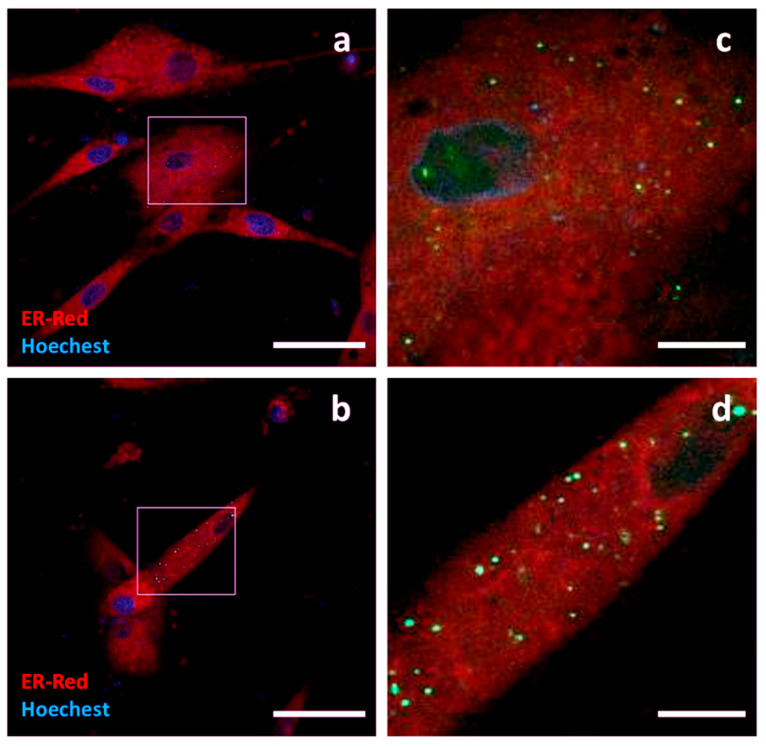
Localization of TSG101-GFP. Confocal micrographs of transfected c-Ad-MSCs overexpressing TSG101-GFP. Nuclei (blue) and endoplasmic reticulum (red) were labelled. TSG101-GFP shows a multifocal pattern in the form of discrete cytoplasmic small spots. Panels (**c**) and (**d**) show higher magnification views of the boxed regions in (**a**) and (**b**). Scale bars: (**a**,**b**) 50 μm, (**c**,**d**) 10 μm.

**Tumour Susceptibility Gene 101 (TSG101)** is a cytoplasmic protein involved in the ESCRT complex, the molecular machinery responsible for the biogenesis of EVs. GFP was fused to the C-terminus of TSG101, cloned from canine cDNA. Only a few c-Ad-MSCs showed fluorescence signals. The intracellular localization of TSG101-GFP was focal, in the form of discrete small spots distributed throughout the cell. Due to the low number of positive cells, it was not possible to perform immunogold labelling (Figure 7).

**Figure 8:**
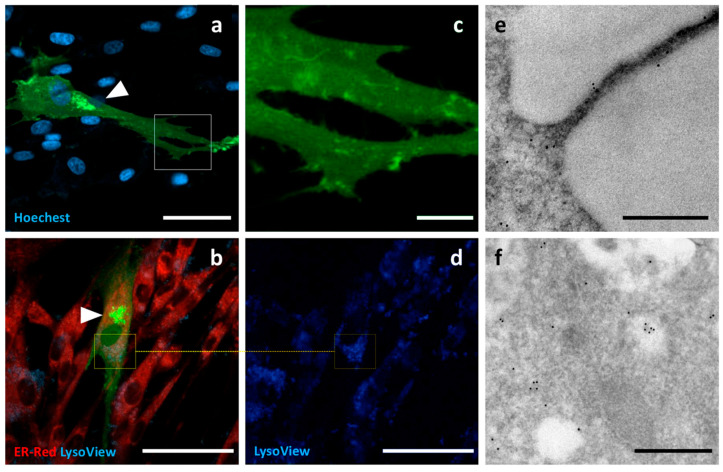
Localization of Palm-GFP. Confocal and TEM micrographs of transfected c-Ad MSCs overexpressing Palm-GFP. Cells were labelled for nuclei (light blue), endoplasmic reticulum (red), and lysosomes (dark blue). Palm-GFP showed a slightly diffuse distribution in the cytoplasm, together with a recurrent strong paranuclear signal, negative for ER or lysosome staining, compatible with the Golgi apparatus (arrowheads) (**a**,**b**). Panel (**c**) shows higher magnification view of the boxed region in (**a**) showing diffuse distribution of the construct. The same field shown in (**b**) is presented in (**d**), highlighting the area outlined by the square and imaged using only the Lysoview filter.Immunogold labelling showed surface-positive protrusions for Palm-GFP (**e**) and intracellular diffuse distribution (**f**). Scale bars: (**a**,**b**,**d**) 50 μm, (**c**) 10 μm, (**e**,**f**) 500 nm.

**Palmitoylated GFP**, a synthetic oligo encoding the N-terminus of canine Lck, was ligated into the GFP-encoding plasmid to obtain a fluorescent probe with a palmitoylation signal in the N-terminus of GFP. c-Ad-MSCs overexpressing Palm-GFP showed diffuse, weak fluorescence and immunoreactivity at TEM. Positive surface-associated signals could also be detected at TEM. Recurrent strong signals from intracellular compartments were observed; they appeared perinuclear, condensed and negative for ER or lysosome staining (Figure 8).

A summary table has been included to visualize tags’ topology, expression, secretion, and localization in transfected c-Ad-MSCs in a synoptic manner (Table 3).

## 4. Discussion

EVs mediate intercellular crosstalk under both normal and pathological conditions. As cell-derived nanostructures, they are living vehicles that hold great promise for transferring functional and therapeutic molecules between cells.

Several studies have investigated the possibility of functionalizing EVs with exogenous biomolecules using tags normally involved in EV biogenesis [9,10,29,30,31,32,33,34,35].

In this study, we engineered MSCs from canine adipose tissue to overexpress GFP tagged with molecules known to be associated with EVs. The aim was to assess the feasibility of protein loading into EVs.

To this end, the canine cDNAs encoding CD63, Syntenin-1, and TSG101 were cloned to obtain chimeric GFP-tagged proteins. Sequencing confirmed the identity of the transcripts and, in the case of Syntenin-1, revealed a splice variant lacking glutamine at position 81 (ΔQ81). As the specific functions of this variant are not yet known, it was not considered in this study. In addition, the palmitoylation signal of Lck as a membrane-anchoring motif was tested.

TSG101-GFP gave the weakest quantitative results. The very low number of transfected c-Ad-MSCs only allowed detection by fluorescence microscopy. Western blotting of cell lysates showed no detectable signal. The low expression efficiency could be due to various factors, including a weak Kozak sequence or the presence of cellular repression systems. Although further evidence is needed, the well-defined multifocal pattern suggests possible localization in multivesicular bodies, as previously observed by Nabhan and colleagues and by Kim and colleagues [36,37]. TSG101 has not previously been used as a tag for EVs engineering, although it is well known in other fields such as virology, where the protein of interest was always fused to its N-terminus [36,37,38,39]. In our study, GFP was fused to the C-terminus of TSG101 to match the topology of the other constructs under study. It is likely that this topological difference negatively affected protein expression and stability.

As for the palmitoylation signal, we observed that it led to a 4-fold enrichment of GFP in EVs compared with untagged GFP. Confocal microscopy revealed that Palm-GFP has a pronounced affinity for the cell membrane. C-Ad-MSCs expressing Palm-GFP also showed diffuse cytoplasmic fluorescence. In addition, juxta-nuclear fluorescent substructures were detected that topographically corresponded to the Golgi apparatus, where a high concentration of the chimeric protein was expected. Post-translational modifications of proteins by the addition of hydrophobic groups—so-called protein lipidation—primarily ensure stable interaction with cell membranes. So far, several types of lipidations have been shown to enhance protein loading into EVs [40]. Palmitoylation is a reversible modification: the lipid anchor is attached to the cysteine residue at the N-term (position 2 or 3) by palmitoyl acyltransferase mainly at the Golgi level and removed by esterase enzymes elsewhere in the cell. For this reason, the process is considered reversible and may therefore reduce EV loading efficiency. At the same time, this reversibility could facilitate cargo detachment from the membrane in recipient cells and subsequent diffusion into the cytoplasm.

Lipid anchors are widely used as tags for EV engineering, especially in EV labelling strategies [41].

Shen and colleagues compared the “budding ability” of different lipid anchors fused to reporter proteins. Based on overexpression in Jurkat cells, they found that myristoylation was the most efficient signal among those tested [42]. Lai and colleagues used the palmitoylation signal to engineer EVs and demonstrated its good performance, as well as its distribution across various cell membrane structures, including exosomes, microvesicles, nanotubes, and membrane protrusions [32].

Syntenin-1 is a 32 kDa cytoplasmic globular protein with multiple functions. Together with Alix and Sindecan, it acts as a protein adaptor involved in the sorting of proteins into exosomes [43]. By binding various cytoskeleton proteins, Syntenin-1 modulates cell adhesion processes and participates in the internalization of certain membrane receptors [44]. Due to its diverse functions, it is not possible to define a single intracellular localization for Syntenin-1. Depending on the interaction and context, it can localize to the cytoskeleton, the nucleus, the perinuclear region, and in association with endosomes and multivesicular bodies [45]. To date, only a few researchers have used Syntenin-1 to engineer EVs. Corso et al. demonstrated that full-length Syntenin-1 efficiently incorporates a reporter protein into EVs [9], while Conceição and colleagues used the N-terminal domain of Syntenin-1 to generate EVs from HEK293 and human MSCs [46]. In both studies, several aspects such as EV composition and EV uptake were analyzed in detail, but the biogenesis of EVs in parental cells was not described. In our study, cloning of the cDNA encoding Syntenin-1 from canine MSC RNA revealed two splice variants: Variant 1 (the main form consisting of 298 amino acids) and Variant 2 (lacking the Glutamine codon at position 81). Overexpression of Syn-GFP in c-Ad MSCs showed widespread cytoplasmic localization and accumulation in small intracellular compartments, likely corresponding to multivesicular bodies. Microscopic observation also revealed numerous fluorescent structures protruding from the cell membrane. Western blotting showed that the full-length chimeric protein (~60 kDa) was mainly detected in the cell lysates, whereas EVs contained both full-length protein and lower-molecular-weight components, possibly arising from post-translational processing or chimeric proteins generated by alternative start codons. A relatively high loading efficiency of the chimeric protein in the EVs was observed, estimated to be 7-fold higher than untagged GFP. Importantly, the enrichment of Syntenin-1 in EVs may influence their cell fate, as Syntenin-containing EVs have been associated with enhanced uptake and intracellular trafficking in recipient cells, suggesting that the broad cellular distribution of Syn-GFP may favour loading of cytosolic cargo and enhance subsequent uptake/trafficking in target cells [43,47].

CD63 or TSPAN30 is a member of the tetraspanin family, a group of biomolecules with four transmembrane domains that are widely recognized as specific markers of EVs. CD63 carries out numerous functions related to the transport of molecules from outside to inside the cell, including the internalization of surface molecules, recycling of substances within the endosomal system, and transport to lysosomes [48]. The use of CD63 as a tag for directing proteins into EVs has been reported in several studies. Koumangoy and colleagues used CD63-GFP expressing cancer cells to investigate the role of exosomes in tumour biology [31].

Stickney and colleagues investigated the feasibility of using tetraspanin family proteins (CD9, CD63, and CD81) to engineer EVs with fluorescent probes and tested different topologies for CD63 [49]. Similar studies were performed by Corso and colleagues, who concluded that CD63 is the most effective tag for modifying EVs [9]. Kojima and Bojar developed an innovative system to produce engineered exosomes using CD63 as a tag [50]. Sung et al. used a pH-sensitive fluorescent reporter system to label EVs from different cancer cell lines using CD63 as a tag, enabling real-time tracking of EV secretion dynamics in space and time [16].

Strohmeier et al. engineered HEK293 cells with genome editing systems to obtain stable lines in which CD63 was constitutively tagged with GFP or HaloTag [15].

McNamara and colleagues described the distribution and co-localization of CD63 and CD81 in lipid rafts and EVs secreted by U-2 osteosarcoma cells [49]. Finally, Jurgielewicz et al. developed a high-throughput screening method (HTS) to study EV uptake using CD63-GFP-labelled EVs derived from HEK293 [14].

Heusermann et al. analyzed the uptake of CD63-GFP or mCherry-labelled EVs derived from HEK293 cells by cultured fibroblasts [51]. All these studies demonstrated that CD63-labelled EVs are efficiently taken up by recipient cells. In addition, some authors reported that overexpression of chimeric CD63 proteins in parental cells leads to increased EV production and release. These properties make CD63 an attractive tool for generating engineered EVs.

In view of the results obtained in our study, our findings suggest that CD63-GFP may represent the most efficient tagging system, even though the estimated secretion index of CD63-GFP in EVs was relatively low compared with the other tested tags. Its overall abundance could reflect both higher protein expression and greater stability/accumulation, while the low secretion index might be influenced by a possible high uptake of CD63-GFP-labelled EVs by non-transfected cells. Indeed, a higher number of fluorescent c-Ad-MSCs were observed by confocal microscopy compared with the other tags.

Western blotting showed that the chimeric protein displayed a banding pattern like that frequently observed when blotting against CD63, likely due to heterogeneous post-translational modifications [45]. The small amount of untagged GFP in the cell lysate and in the extract of EVs can be interpreted as a possible product of cleaved precursor or another ATG start codon during the translation process.

The intracellular localization of CD63-GFP in c-Ad-MSCs is consistent with previous findings in cultured human and murine cells. In our study, CD63-GFP was mainly detected in the perinuclear region, associated with membrane-bound intracellular compartments morphologically compatible with endosomes and multivesicular bodies, which are typically involved in exosome biogenesis.

Given that CD63-enriched EVs are typically processed through endosomal pathways and can influence cargo localization and uptake, it is reasonable to hypothesize that therapeutic molecules loaded via CD63-tagged EVs might follow a comparable biodistribution and functional pattern in recipient cells. Such a scenario would imply that CD63 not only contributes to efficient EV loading but could also indirectly shape the availability and activity of therapeutic cargo [13,52]. However, this remains a hypothesis, and further experimental studies will be needed to confirm these assumptions and to better elucidate the mechanisms involved. Collectively, these features indicate that CD63-EVs may play a unique role in intercellular communication, with implications for both physiological processes and therapeutic applications.

## 5. Conclusions

In recent years, interest in EVs has grown considerably due to their diverse applications in the biomedical field, ranging from diagnostics to therapy. Currently, great expectations are placed on the use of EVs as drug carriers, especially for the delivery of therapeutic nucleic acids or proteins.

The aim of this study was to develop a system for producing engineered EVs derived from a primary cell type of therapeutic interest in veterinary medicine. Specifically, we used MSCs from the adipose tissue of dogs, an animal species in which both cell-based and cell-free therapies are already at an advanced stage of application. Their efficacy and safety have been demonstrated in many applications in companion animals with very encouraging results. Although this study was performed using MSCs derived from a single donor, donor-to-donor variability is expected to have a limited impact, as the efficiency of the constructs depends mainly on species-specific sequence homology rather than individual genetic differences.

The growing interest in the MSC secretome and EVs represents the natural progression of advanced therapies, both because of their inherent biological activity and their intriguing potential as drug delivery systems.

An increasing number of studies have demonstrated the efficacy and feasibility of cell-free therapies in veterinary medicine [53]. Although the road to large-scale production of EVs is still long, many companies are showing interest in developing EV-based therapeutic products. Engineering of EVs is another rapidly expanding research area driven by the discovery that EVs can serve as vehicles for transporting various types of therapeutic molecules.

To summarize, the novelties presented in this study are multifaceted. First, we used primary cells that are already employed in the treatment of dog diseases, for which the efficacy and safety of the derived EVs have been demonstrated. Many research groups have investigated strategies to engineer human and murine cells to modify the protein content of EVs. However, most of these studies have relied on immortalized cell lines, mainly HEK293 cells. The development of EV-based delivery systems requires a shift toward safer, clinically relevant cell sources for in vivo applications. Second, our findings suggest, for the first time in dogs, that CD63 might be the most effective tag for engineering c-Ad-MSC-derived EVs. The combination of robust loading efficiency and well-confined intracellular localization appears to support both reliable cargo incorporation and spatially regulated release. While the secretion index might suggest a preferential role for Syntenin-1, the consistently higher abundance of CD63 in the lysate supports the hypothesis of significant post-secretion uptake. Taken together, these features indicate that CD63 could be particularly suitable for advanced engineering strategies aimed at loading and delivering therapeutic biomolecules into cAd-MSC-EVs. Further studies aimed at better deciphering its functional contribution and identifying which domains of the molecule are involved in vesicle trafficking could offer valuable insights into EVs bioengineering. Nevertheless, it is important to note that the other tags tested—particularly Syntenin-1— also produced encouraging results in terms of EV loading. These findings confirm that a protein of interest is incorporated into EVs more efficiently when fused to EV-associated proteins, opening the possibility of using different tags to optimize ca-nine EV engineering for specific applications.

Third, we used MSCs derived from an animal species that is not only relevant as a target for potential personalized therapy with functionalized EVs but also represents a model for the development of human therapies that is much closer and more reliable than traditional murine models.

In this specific context, the human protein sequences of CD63, Syntenin-1, and TSG101 show higher identity and similarity to their canine orthologs than those of mice (Table S1). Last but not least, from a purely technological standpoint, we demonstrated the feasibility of a method to generate GFP-tagged MSC-EVs, which could serve as a valuable tool for studying vesicle biogenesis and for investigating EV trafficking. Our long-term goal is to produce therapeutic MSC-EVs that can be used in animals with spontaneous diseases and serve as reliable models for human pathologies.

## Figures and Tables

**Table 1:** List of genes encoding for selected tags and localization site in EVs.

Protein Name	Gene ID	Protein Size	EVs Localization	Specific EVs Motif/Domain
**Lck** (Tyrosine-protein kinase Lck)	478151	509 aa/58 kDa	membrane anchor	Palmitoylation signal in N-term (MGCSCSSNPE)
**CD63**	474391	238 aa/25.6 kDa	transmembrane	Unknown
**SDCBP** (Syntenin-1)	482977	298 aa/32 kDa	luminal	(LYPXnL)
**TSG101** (Tumour susceptibility gene 101)	485406	391 aa/44 kDa	luminal	(PTAP)

**Table 2:** List of primers and oligos.

Gene of Interest	Sequence Expected Size (bp)	Primer/Oligo Sequences	Thermal Conditions
**CD63**	740	F: 5′-GGCAAGCTTCCATGGCGGTGGAAGG-3′ R: 5′-GAGAGTCGACCCCTACATGACTTCATAGCCAC-3′ for cloning in pBlueScriptII-SK plasmid	98° × 10″ 65° × 15″ 72° × 1′
**TSG101**	1255	F: 5′-CCCTAAGCTTGCGGTGACTGGAGTGG-3′ R: 5′-GCTTTAAGTCGACCTCAATCTCCAGCTGAT-3′ for cloning in pBlueScriptII-SK plasmid	98° × 10″ 57° × 20″ 72° × 1′10″
**SDCBP (Syntenin-1)**	1021	F: 5′-AAAAGGTACCTCTGCAAAAATGTCTCTCTACCCA-3′ R: 5′-AAAAGTCGACTGGCTCCTGGAAAGCTTCA-3′ for cloning in pBlueScriptII-SK plasmid	98° × 10″ 60° × 15″ 72° × 1′
**CD63**	735	F: 5′-GGCAAGCTTCCATGGCGGTGGAAGG-3′ R: 5′-AAAACGTCGACATGACTTCATAGCC-3′ for subcloning in pTagGFP2-N plasmid	98° × 10″ 65° × 15″ 72° × 1′
**TSG101**	1217	F: 5′-CCCTAAGCTTGCGGTGACTGGAGTGG-3′ R: 5′-AAAACTCGAGTAGAGGTCACTGAGACC-3′ for subcloning in pTagGFP2-N plasmid	98° × 10″ 57° × 20″ 72° × 1′10″
**SDCBP (Syntenin-1)**	921	F: 5′-AAAAGTCGACTCTGCAAAAATGTCTCTCTACCC-3′ R: 5′-AAAACGTCGACACCTCAGGAATGGTGTG-3′ for subcloning in pTagGFP2-N plasmid	98° × 10″ 60° × 15″ 72° × 1′
**Palm sequence**	45–37	Sense: 5′-AGCTTGCCATGGGCTGTAGCTGCAGCTCAAACCCTGAAGCGGTAC-3′ Antisense: 5′-CGCTTCAGGGTTTGAGCTGCAGCTACAGCCCATGGCA-3′	N.A. (denaturation at 80 °C and slow annealing in cooling water)

**Table 3:** **Synoptic view of EVs related tags.** Summary table showing the membrane topology of GFP fusion constructs, their relative abundance, and the intracellular localization. The “Tag and relative topology” row illustrates the predicted membrane orientation of each fusion protein, indicating whether the GFP moiety faces the vesicle lumen, the cytosolic side, or both. GFP abundance in cell lysates and EVs is expressed relative to untagged GFP. In the last row, the green schematic highlights the observed distribution of fluorescence corresponding to the localization of each GFP signal.

Tag and Relative Topology	Palmitoylated-GFP 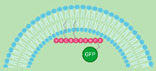	CD63-GFP 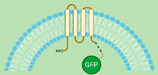	Syntenin1-GFP 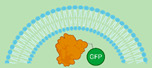	TSG101-GFP 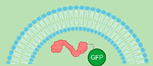
**GFP relative abundance** **in cell lysates** (compared to untagged GFP)	2 fold	5 fold	1,6 fold	Not detected
**GFP relative abundance** **in Evs** (compared to untagged GFP)	4 fold	6 fold	7 fold	Not detected
**Intracellular** **Localization**	Cell membrane and Golgi apparatus 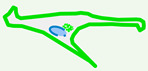	Cell membrane, endosomes, endoplasmic reticulum 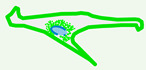	Cytoplasm 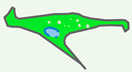	Focal spots, not defined 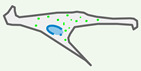

## Data Availability

The original contributions presented in this study are included in the article/Supplementary Material. Further inquiries can be directed to the corresponding author.

## Supplementary Materials

The following supporting information can be downloaded at: https://www.mdpi.com/article/10.3390/pharmaceutics17111435/s1; Table S1: Comparison of protein sequence identity and similarity of CD63, Syntenin-1, and TSG101 among humans, dog, and mouse species.

## Abbreviations

The following main abbreviations are used in this manuscript:

EVs	Extracellular vesicles
Ex	Exosomes
MVBs	Multivesicular bodies
MVs	Microvesicles
ABs	Apoptotic bodies
ESCRT	Endosomal sorting complex required for transport
MSCs	Mesenchymal Stromal Cells
c-Ad-MSCs	Canine adipose-derived MSCs
GFP	Green Fluorescent Protein
OVUD	Veterinary University Hospital
DMEM	Dulbecco’s modified Eagle Medium
SVF	Stromal Vascular Fraction
FBS	Fetal Bovine Serum
ISCT	International Society for Cell and Gene Therapy
DPBS	Dulbecco’s Phosphate-Buffered Saline
