# Supplementary material for: Hacking Extracellular Vesicles: Using Vesicle-Related Tags to Engineer Mesenchymal Stromal Cell-Derived Extracellular Vesicles
Source: Pharmaceutics. 2025 Nov 6;17(11):1435. doi: 10.3390/pharmaceutics17111435 (PMC12655496; doi:10.3390/pharmaceutics17111435)
Supplement: Supplementary file 1 [file pharmaceutics-17-01435-s001.zip › pharmaceutics-3831269-supplementary.pdf]

pharmaceutics

Table S1. Comparison of protein sequence identity and similarity of CD63, Syntenin-1, and TSG101 among humans, dog, and mouse species.

Sequence ID	DOG	HUMAN	MOUSE
CD63 (Tspan30)	XP_038534846.1	NP_001244318.1	NP_001036045.1
SDCBP (Syntenin-1)	XP_038297152.1	NP_001091697.1	NP_001007068.1
TSG101	XP_038286593.1	NP_006283.1	NP_068684.1
**CD63**			
	Identity	Similarity	
Canine vs. Human	214/238 (90%)	223/238 (93%)	
Human vs. Murine	189/238 (79%)	210/238 (88%)	
**SDCBP (Syntenin-1)**			
	Identity	Similarity	
Canine vs. Human	282/298 (95%)	292/298 (97%)	
Human vs. Murine	270/299 (90%)	287/299 (95%)	
**TSG101**			
	Identity	Similarity	
Canine vs. Human	382/391 (98%)	384/391 (98%)	
Human vs. Murine	370/391 (95%)	381/391 (97%)	

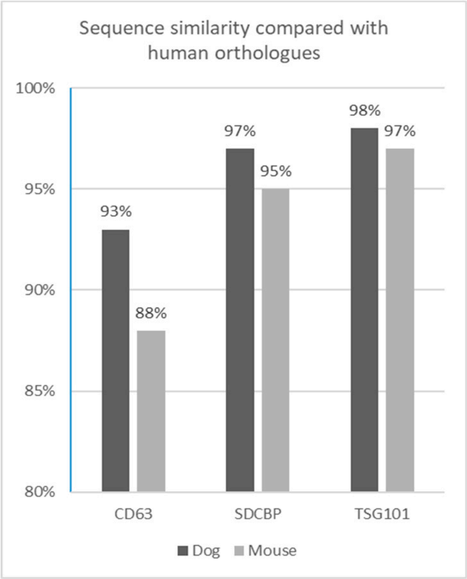
Sequence similarity compared with human orthologues
100%
95%
90%
85%
80%
93%
88%
97%
95%
98%
97%
CD63
SDCBP
TSG101
Dog
Mouse

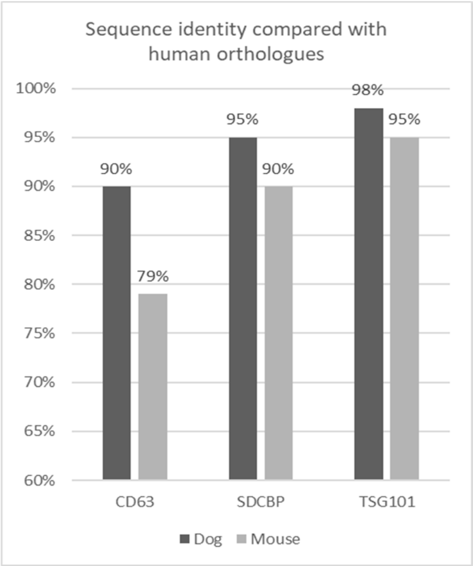
Sequence identity compared with human orthologues
100%
95%
90%
85%
80%
75%
70%
65%
60%
90%
79%
95%
90%
98%
95%
CD63
SDCBP
TSG101
Dog
Mouse
